# DNA Supercoiling Regulates the Motility of *Campylobacter jejuni* and Is Altered by Growth in the Presence of Chicken Mucus

**DOI:** 10.1128/mBio.01227-16

**Published:** 2016-09-13

**Authors:** Claire Shortt, Eoin Scanlan, Amber Hilliard, Chiara E. Cotroneo, Billy Bourke, Tadhg Ó Cróinín

**Affiliations:** a School of Biomolecular and Biomedical Science, University College Dublin, Belfield, Dublin, Ireland; b National Children’s Research Centre, Our Lady’s Hospital for Sick Children, Crumlin, Dublin, Ireland

## Abstract

*Campylobacter jejuni* is the leading cause of bacterial gastroenteritis in humans, but relatively little is known about the global regulation of virulence factors during infection of chickens or humans. This study identified DNA supercoiling as playing a key role in regulating motility and flagellar protein production and found that this supercoiling-controlled regulon is induced by growth in chicken mucus. A direct correlation was observed between motility and resting DNA supercoiling levels in different strains of *C. jejuni*, and relaxation of DNA supercoiling resulted in decreased motility. Transcriptional analysis and Western immunoblotting revealed that a reduction in motility and DNA supercoiling affected the two-component regulatory system FlgRS and was associated with reduced FlgR expression, increased FlgS expression, and aberrant expression of flagellin subunits. Electron microscopy revealed that the flagellar structure remained intact. Growth in the presence of porcine mucin resulted in increased negative supercoiling, increased motility, increased FlgR expression, and reduced FlgS expression. Finally, this supercoiling-dependent regulon was shown to be induced by growth in chicken mucus, and the level of activation was dependent on the source of the mucus from within the chicken intestinal tract. In conclusion, this study reports for the first time the key role played by DNA supercoiling in regulating motility in *C. jejuni* and indicates that the induction of this supercoiling-induced regulon in response to mucus from different sources could play a critical role in regulating motility *in vivo*.

## IMPORTANCE

Although *Campylobacter jejuni* is the leading cause of bacterial gastroenteritis, very little is understood about how this pathogen controls the expression of genes involved in causing disease. This study for the first time identifies DNA supercoiling as a key regulator of motility in *C. jejuni*, which is essential for both pathogenesis and colonization. Altering the level of DNA supercoiling results in changes in motility levels, as well as changes in the expression of genes involved in flagellar gene regulation. Furthermore, spontaneous clones of the organism with different motility profiles have altered DNA supercoiling levels. Finally, mucus was identified as a key stimulator of changes in DNA supercoiling, and it was shown that mucus from different sites in the chicken intestine induced different levels of DNA supercoiling. In conclusion, this study implicates DNA supercoiling as a key regulator of motility in *C. jejuni in vivo* during colonization of the mucus layer.

## INTRODUCTION

*Campylobacter jejuni* is the most common cause of bacterial gastroenteritis worldwide. The organism naturally colonizes the avian intestinal tract, where the bacteria can be found in high numbers without causing disease, leading to chicken meat being a key reservoir of infection for this pathogen (1). In contrast, infection of humans typically leads to an acute infection associated with an invasive phenotype and enterocolitis. Although a number of adhesins and invasins have been identified, the exact nature of the interaction between this pathogen and the host cell is still unclear (2). However, one factor that has been proven conclusively to play a role during colonization, as well as pathogenesis, is the presence of flagella. *Campylobacter* flagellar mutants colonize chickens between 100- and 1,000-fold less efficiently than wild-type (WT) bacteria (3), and motility and functional flagella have been implicated as being essential for infection of the human intestine.

*C. jejuni* produces a single flagellum at each pole and contains a flagellar expression system that has recently been revealed to contain several novel components unique to epsilonproteobacteria (4). The complex flagellar structure of *C. jejuni* plays a role in motility but has also been reported to function as a protein export apparatus for the secretion of factors involved in invasion of host epithelial cells (5, 6). Many of the structural and regulatory components of the flagellar apparatus have now been characterized (7). Flagellar biosynthesis is tightly controlled through two sigma factors (σ^28^ and σ^54^) that are involved in the sequential expression of different sets of genes for the proper assembly of the flagellar structure (8). In addition to these sigma factors, a variety of studies have highlighted the importance of a novel two-component regulatory system (FlgRS) that is critical for the initiation of transcription of flagellar structural genes (8, 9). FlgR is a response regulator that lacks a DNA binding domain but upon phosphorylation leads to the activation of σ^54^ and subsequent transcription of genes under the regulation of this sigma factor (10, 11). FlgS is found in the cytoplasm and is not membrane associated, as is commonly observed for other sensor kinases (12). The exact mechanism of activation and autophosphorylation of FlgS is currently unknown, but recent studies have linked this histidine kinase to the flagellar export apparatus, placing this two-component regulatory system at a key point in the regulatory cascade between the initial formation of the flagellar export apparatus and downstream flagellar genes under the control of σ^54^ and σ^28^ (12, 13). Phase variation has been identified as playing a role in flagellar regulation, and recent studies have characterized phase variation in FlgS, FlgR, and other genes involved in motility (14–16). Further exploration of the role and regulation of the flagellar apparatus in protein export and motility will be key to understanding how *C. jejuni* colonizes chicken and human intestines.

In contrast to other gastrointestinal pathogens, relatively little is known about the global regulation of virulence genes in *C. jejuni*. Of particular note is the lack of global gene regulators and the presence of only three known sigma factors (8). There is also a notable absence of homologues of many of the nucleoid-associated proteins that have been shown to play an important role in global gene regulation in other members of the *Proteobacteria*. The critical role that these proteins have been shown to play in global regulation of genes in *Salmonella* (17–19) and *Escherichia coli* (20, 21) suggests that gene regulation across the chromosome in *C. jejuni* is likely to differ substantially from that of these better characterized pathogens.

In recent years, a variety of studies have demonstrated the key role that DNA supercoiling plays in gene regulation in a variety of pathogens (22–27). Changes to the DNA topology of the bacterial cell are predominantly a result of two competing enzymes, DNA topoisomerase I (TopA), which relaxes DNA, and DNA gyrase (GyrA/GyrB), which is responsible for introducing negative supercoiling. The regulation of the transcription of these genes maintains a homeostatic balance of DNA supercoiling that benefits the cell (28). DNA supercoiling is sensitive to various environmental conditions, and pathogenic bacteria can exploit this system by using supercoiling-sensitive promoters to regulate virulence genes in response to these changes in the environment (29–31).

One of the key *in vivo* environments faced by *C. jejuni* is the mucus layer overlaying epithelial cells in the intestine of both chickens and humans. Chicken mucus has been shown to have a dramatic effect on the pathogenicity of *C. jejuni in vitro*, with exposure to chicken mucus reducing the ability of the bacteria to invade primary epithelial cells (32). In contrast, no such inhibition was observed using mucous isolated from humans (32). A further study identified mucin as the component which inhibited invasion and showed that mucin from different sites in the chicken gut resulted in different levels of inhibition (33). More recent studies have identified specific mucins in chicken mucus to which *C. jejuni* displays a tropism, suggesting that mucus and the mucins it contains could act as a stimulus to alter the regulation of virulence genes (34).

Although several studies have characterized changes in the transcriptome of *C. jejuni* in response to a variety of environmental stimuli, relatively little is known about how these changes are regulated at a molecular level (35, 36). Interestingly, one study has revealed that the major outer membrane protein (MOMP) of *C. jejuni* may be regulated through DNA supercoiling (37). Treating bacteria with subinhibitory levels of novobiocin to reduce GyrB activity and relax DNA supercoiling induced a reduction in the expression of the MOMP protein in *E. coli* and *C. jejuni*. Another study reported that in the closely related bacterium *Helicobacter pylori*, a variety of flagellar genes were shown to be regulated by changes in DNA supercoiling (38). In this study, we identify the key role of DNA supercoiling in the regulation of motility in *C. jejuni*. We reveal a direct correlation between DNA supercoiling and motility, report that changes in DNA supercoiling lead to divergent expression of the FlgRS two-component regulatory system, and reveal for the first time that chicken mucus from different sites in the chicken intestine can have different effects on DNA supercoiling levels and, thus, dictate flagellar gene expression and the functionality of the flagella.

## RESULTS

### Correlation between motility and DNA supercoiling in laboratory strains of *C. jejuni*.

**FIG 1:**
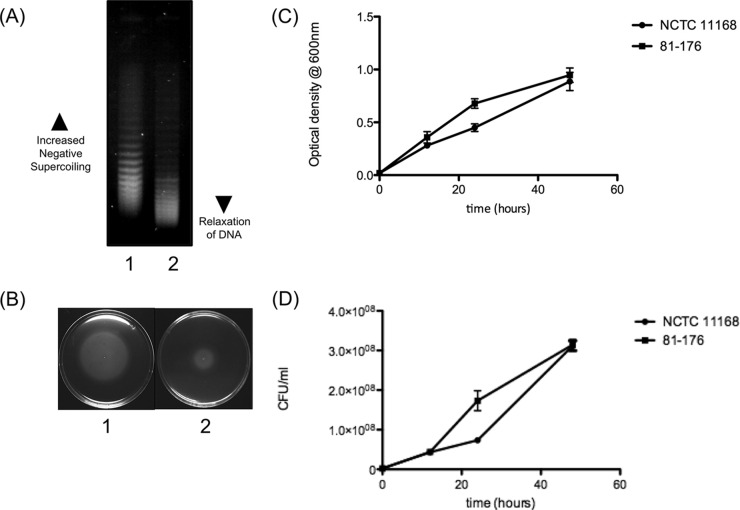
The correlation between DNA supercoiling and motility. (A) DNA supercoiling levels of *C. jejuni* laboratory strains. Lane 1, strain NCTC 11168; lane 2, strain 81-176. (B) Motilities of *C. jejuni* laboratory strains. Plate 1, NCTC 11168; plate 2, 81-176. (C) Optical densities of NCTC 11168 and 81-176 over a 48-h period. (D) CFU/ml of NCTC 11168 and 81-176 over a 48-h period. Error bars show standard deviations.

To investigate the resting DNA supercoiling levels of *C. jejuni* strains NCTC 11168 and 81-176, both strains were transformed with the shuttle vector pRY107. When the plasmid topoisomers are separated on a gel containing 10-µg/ml chloroquine, the more relaxed topoisomers migrate further in the gel, whereas the more negatively supercoiled topoisomers are retained higher in the gel. Chloroquine gel analysis revealed that the plasmid isolated from NCTC 11168 was markedly more negatively supercoiled than the plasmid isolated from strain 81-176 (Fig. 1A). When the same strains were then tested for motility (Fig. 1B), the more negatively supercoiled strain (NCTC 11168) was also notably more motile than the more relaxed strain (81-176). This difference in the motility of each strain could not be attributed to growth differences, as both strains displayed similar growth curves (Fig. 1C and D). These initial results suggested a correlation between increased negative supercoiling and increased motility in *C. jejuni* strains.

### DNA supercoiling and motility in strains isolated from humans and chickens and spontaneous subpopulations.

**FIG 2:**
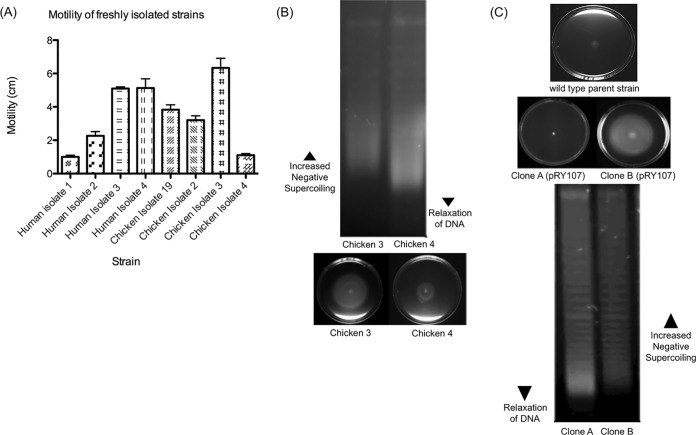
The stability of the DNA supercoiling-motility correlation is preserved within spontaneous subpopulations and within *C. jejuni* isolates from humans and chickens. (A) A panel of human and chicken isolates was assayed for motility levels (*n* = 3). Error bars show standard deviations. (B) Two *C. jejuni* chicken isolates were tested for DNA supercoiling-motility correlation. The more motile strain (chicken 3) displayed a more negatively supercoiled profile than the less motile strain (chicken 4). (C) Motility of a wild-type *C. jejuni* isolate which was transformed with the plasmid pRY107. Random colonies selected after the natural transformation and motility plates of clone A and clone B are shown. The differing motilities of clone A and clone B correlate with their DNA supercoiling levels, with the more motile clone B showing a more negatively supercoiled profile than the less motile clone A.

To explore further the correlation between DNA supercoiling levels and motility in *C. jejuni*, a variety of strains isolated from chickens and humans were tested for motility (Fig. 2A). Strains with various levels of motility were isolated from both chickens and humans. DNA supercoiling was consistently correlated with motility levels in each strain, with more motile strains showing more negative supercoiling and less motile strains showing more relaxed DNA topology (Fig. 2B). These data suggested that regardless of the source of the strain, the observed correlation between DNA supercoiling and motility was still evident.

Interestingly, it was also observed that after transformation with the shuttle vector, some strains would occasionally spontaneously give rise to clones that had different motility profiles. For example, WT strains with low motility after transformation with the plasmid occasionally gave rise to clones that were more motile (Fig. 2C). However, upon testing the individual clones, it was observed that the more motile clones consistently displayed a more negatively supercoiled DNA topology, while the less motile clones displayed a more relaxed supercoiling profile. These results suggested not only that DNA supercoiling levels and motility levels were correlated across a wide variety of strains of *C. jejuni* but that spontaneous clones with different motility levels were being generated, which in turn displayed different DNA topologies. This observation, coupled with the observations in the laboratory strains and the strains isolated from humans and chickens, suggested a strong correlation between DNA supercoiling levels and motility in *C. jejuni*.

### Altering DNA supercoiling levels alters motility levels in *C. jejuni*.

**FIG 3:**
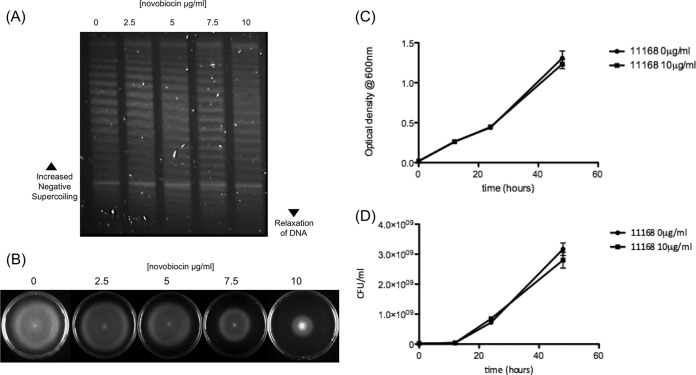
DNA relaxation reduces motility but does not affect viability. (A) The plasmid pRY107 isolated from NCTC 11168 was dose dependently relaxed by growth in subinhibitory levels of novobiocin. (B) Novobiocin-induced DNA relaxation results in a significant reduction in motility. (C) Growth in the presence of 10-µg/ml novobiocin does not affect the optical density of NCTC 11168 over the course of a growth curve. (D) Growth in the presence of 10-µg/ml novobiocin does not alter CFU/ml of NCTC 11168 over the course of a growth curve. Error bars show standard deviations.

In order to test whether pharmacological alteration of DNA supercoiling would change DNA motility levels, bacteria were grown in the presence of novobiocin. Growth in the presence of subinhibitory levels of novobiocin has previously been shown to lead to more relaxed DNA topology, caused by decreased gyrase activity due to a reduction in the ability of GyrB to utilize ATP. For these experiments, *C. jejuni* NCTC 11168 was chosen, as it has high levels of motility and negative supercoiling. Chloroquine gel analysis revealed a progressive increase in more relaxed topoisomers and a reduction in more negatively supercoiled topoisomers following novobiocin treatment (Fig. 3A). This reduction in negative supercoiling was also shown to correlate with a decrease in the motility of the strain (Fig. 3B). A dose-dependent decrease in motility was observed in strains inoculated into motility plates containing increasing concentrations of novobiocin, with significantly lower motility observed at a concentration of 10 µg/ml. To confirm that this observation was not due to an inhibition of growth at these concentrations of novobiocin, the bacteria were grown in Mueller-Hinton (MH) broth at 10 µg/ml of novobiocin, and no significant difference was observed in growth (Fig. 3C and D). Other motile strains treated with novobiocin showed similar reductions in motility (data not shown). These results showed that not only did DNA supercoiling levels and motility correlate but motility could be directly reduced by relaxation of DNA supercoiling.

### Identification of flagellar genes affected by DNA supercoiling levels.

**FIG 4:**
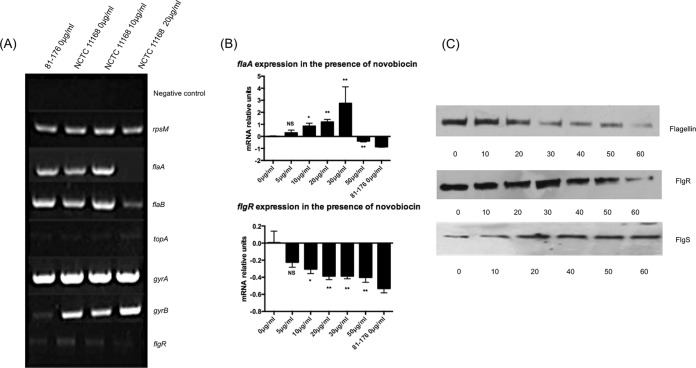
DNA relaxation reduces motility through an alteration of flagellar transcription and protein expression. (A) RT-PCR of flagellar and topoisomerase genes from strain 81-176 and strain NCTC 11168 grown with or without novobiocin (0, 10, or 20 µg/ml). (B) qPCR of *flaA* and *flgR* confirms the RT-PCR results. Changes in gene expression for each gene are shown in comparison to the resting levels in strain NCTC 11168 grown without novobiocin. Changes in gene expression in strain NCTC 11168 in the presence and absence of novobiocin are noted as being significant if they had a *P* value of <0.05 (*), very significant at a *P* value of <0.01 (**), or not significant (NS). Error bars show standard deviations. (C) Western blots of protein lysates of strain NCTC 11168 grown with increasing concentrations of novobiocin using flagellin antiserum, FlgR antiserum, and FlgS antiserum.

As DNA supercoiling appeared to influence motility, reverse transcription-PCR (RT-PCR) was used to examine the effect of DNA relaxation on the transcription of a variety of genes known to be involved in DNA supercoiling and motility. The more negatively supercoiled strain NCTC 11168 was grown under increasing concentrations of novobiocin, and gene expression was compared to that observed in the more relaxed strain 81-176 (Fig. 4A). The ribosomal gene *rpsM* was used as a control gene, as no difference was observed in the expression of this gene between the two strains or upon relaxation of DNA supercoiling in strain NCTC 11168. Similarly, no difference was observed in *gyrA*, suggesting that this gene was also unaffected by changes in DNA supercoiling levels. *gyrB* expression was higher in the more supercoiled NCTC 11168 strain than in the more relaxed 81-176 strain, suggesting that differences in the baseline transcription levels of this subunit of the gyrase could account for the strain-dependent difference in DNA supercoiling levels. In addition, relaxation of DNA supercoiling in strain NCTC 11168 led to an increase in *gyrB* transcription, suggesting that, as observed in other bacteria, the *gyrB* promoter in *C. jejuni* is induced by more relaxed DNA topology. The transcription of *topA* in both strains and under all conditions was low, as would be expected in log-phase bacteria (28, 39). The transcription levels of the genes encoding the flagellar subunits *flaA* and *flaB* were similar in both strains. However, the transcriptional activity of these genes following relaxation of DNA supercoiling showed a biphasic response, with an initial increase followed by a decrease in transcription. Finally, the transcription of the gene encoding the flagellar regulatory protein FlgR was observed to be lower in the relaxed strain 81-176 and was dose dependently decreased upon DNA relaxation of strain NCTC 11168 in the presence of novobiocin. These results suggested that some flagellar genes were being regulated by DNA supercoiling, with a clear decrease in the transcription of *flgR* and an aberrant effect on *flaA* and *flaB* expression which resulted in an initial increase and then a significant decrease in expression. All experiments were then repeated using quantitative real time PCR (qPCR), and the results for *flgR* and *flaA* confirmed these initial observations by RT-PCR (Fig. 4B).

To confirm that the observed changes in gene transcription resulted in similar alterations at a protein level, the presence of the encoded proteins was analyzed by Western immunoblotting. Strain NCTC 11168 was grown under increasing concentrations of novobiocin, and Western blots were probed with antibodies against flagellin, FlgR, and FlgS. A gradual decrease in flagellin was observed upon relaxation of DNA supercoiling, coupled with reduced levels of FlgR. However, when the same blots were probed with an antibody to FlgS, the histidine kinase known to phosphorylate FlgR, a dose-dependent decrease in protein levels was observed (Fig. 4C). These results together suggested that, although the regulation of flagellar genes was being affected, this was not a simple decrease in the expression of all genes and that relaxation of DNA supercoiling was leading to aberrant expression of the FlgRS two-component regulatory system, with an increase in FlgS expression and a decrease in FlgR expression.

### Screening for evidence of a role for phase variation in supercoiling-induced differential expression of FlgR and FlgS.

Recent studies have documented the role of specific homopolymeric A tracts and other genetic variations in distinct sites in *flgR* and *flgS*, as well as *motA*, in phase variation (14–16). To investigate whether these alterations could explain the differences observed after altering supercoiling levels, the relevant regions were sequenced from bacteria grown in the presence and absence of novobiocin (see Fig. S1 in the supplemental material). No evidence of changes in the previously described regions could be identified. The relevant regions were also sequenced from the two spontaneous clones previously shown to have altered DNA supercoiling and altered motility, and again, no change was observed in the specific regions previously identified as being involved in phase variation of *flgR*, *flgS*, or *motA* (see Fig. S2).

### Analysis of the effect of relaxation of DNA supercoiling on the flagellar structure.

**FIG 5:**
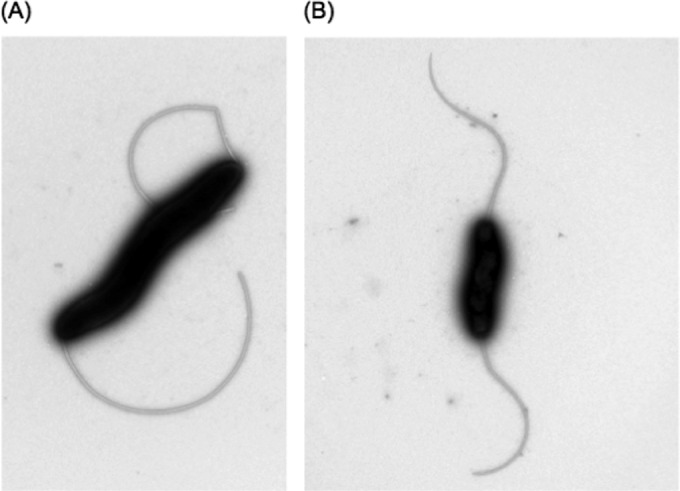
Transmission electron microscopy of NCTC 11168 in the absence and presence of novobiocin. No observable difference was seen between more motile bacteria with more supercoiled DNA (A) and bacteria treated with novobiocin, which were less motile and had a more relaxed DNA topology (B).

Although DNA supercoiling appeared to influence the transcription of flagellar genes, it was unclear whether the flagellar structure was being completely inhibited or affected in a more subtle way. To test whether the altered expression observed for flagellar genes resulted in an effect on the flagellar structure, transmission electron microscopy was carried out on NCTC 11168 bacteria cultured in the presence and absence of novobiocin. In total, over 50 images were acquired and analyzed, and there were no observable changes in the production of flagella between NCTC 11168 bacteria treated with novobiocin and NCTC 11168 bacteria cultured in MH medium only (Fig. 5). These results suggested that, although the transcription of flagellar genes and the consequent expression of proteins was being affected, the decrease in motility was not due to a lack of the flagellar structure.

### The effect of porcine mucin on motility and DNA supercoiling.

**FIG 6:**
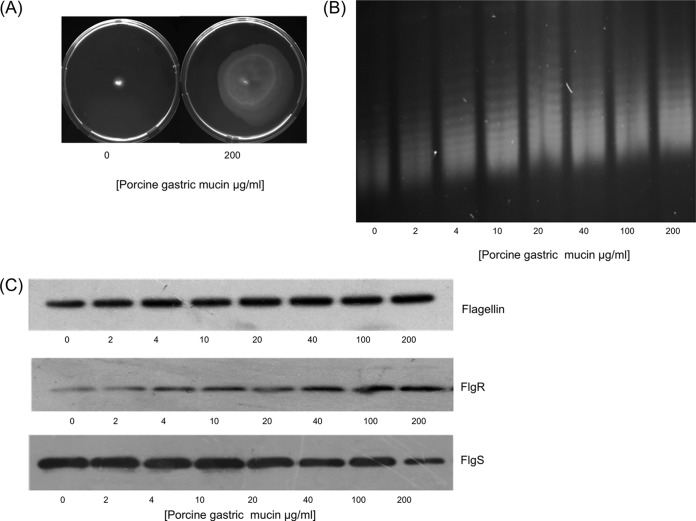
Growth in porcine gastric mucin induces increased negative supercoiling and motility and alters flagellar protein expression in strain 81-176. (A) Growth of 81-176 in motility agar plates containing 200-µg/ml porcine gastric mucin increases motility. (B) Increasing concentrations of porcine gastric mucin lead to increased negative supercoiling. (C) Porcine mucin leads to increased levels of flagellin and FlgR but decreased levels of FlgS.

The effect of novobiocin on DNA supercoiling, motility, and the expression of flagellar genes showed that a relaxation of DNA topology could significantly reduce the motility of *C. jejuni*. To test whether the growth of *C. jejuni* under conditions commonly encountered *in vivo* could affect DNA supercoiling and motility, growth in the presence of porcine mucin was tested for its effect on the motility of the less motile strain, 81-176. Growth in the presence of 200-µg/ml porcine mucin led to a significant increase in the motility of 81-176 (Fig. 6A). This suggested that growth in the presence of porcine mucin was having an opposite effect to that observed with growth in the presence of novobiocin. When DNA supercoiling levels were analyzed, this was confirmed, as a dose-dependent increase in negative supercoiling was observed (Fig. 6B). To test whether similar changes in the expression of flagellar proteins were observed, Western immunoblotting was carried out on protein samples from 81-176 bacteria grown in the presence of increasing concentrations of mucin (Fig. 6C). As predicted, this dose-dependent increase in negative supercoiling also led to a dose-dependent increase in flagellin expression. Interestingly, this increase in motility and increase in negative supercoiling also led to an effect on the expression of the FlgRS two-component regulatory system, but under these conditions, FlgR levels increased, whereas levels of FlgS decreased. These results revealed that not only could increased negative supercoiling lead to greater motility but the expression levels of FlgR and FlgS were directly opposite to those observed when motility was decreased by the relaxation of DNA supercoiling.

### The effect of chicken mucus on DNA supercoiling and flagellar protein expression.

**FIG 7:**
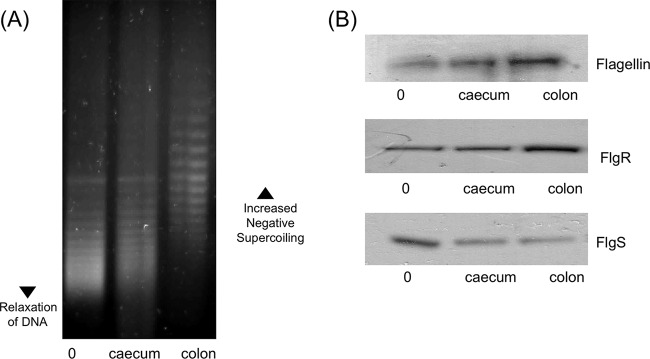
Growth in the presence of chicken mucus promotes motility and increased negative DNA supercoiling, and this effect is more pronounced with colonic mucus than with mucus from the cecum. (A) Growth in the presence of 100 µg/ml of chicken cecal mucus subtly increases negative DNA supercoiling levels, but colonic chicken mucus significantly increases negative supercoiling levels. (B) Growth in the presence of 100 µg/ml of chicken colonic or cecal mucus leads to increased expression of FlgR and decreased expression of FlgS, which is more pronounced with colonic than with cecal mucus.

The effects of novobiocin and porcine gastric mucin on DNA supercoiling levels and motility showed that alteration of DNA supercoiling could either increase or decrease the motility of *C. jejuni* strains. Finally, it was decided to test whether this regulation of motility through DNA supercoiling could be affected by growth under conditions more representative of *in vivo* conditions. To test this hypothesis, mucus was isolated from the colon and cecum of chickens, and strain 81-176 carrying the pRY107 plasmid was grown in the presence and absence of this mucus. There was a slight increase in negative DNA supercoiling among bacteria exposed to mucus from the cecum. However, growth in mucus isolated from the colon of chickens resulted in a pronounced increase in negative DNA supercoiling (Fig. 7A). This effect of chicken mucus on supercoiling levels was also mirrored in the expression of flagellar proteins (Fig. 7B), with a moderate increase in FlgR and flagellin expression in cecal mucus but a much greater increase in colonic mucus. FlgS expression was decreased in bacteria grown in the presence of both colonic and cecal mucus. These results showed that not only does growth in the presence of chicken mucus affect DNA supercoiling levels and flagellar protein expression but the site from which the mucus is isolated can have a profound effect on the level of increase in negative supercoiling and subsequent gene expression.

## DISCUSSION

DNA supercoiling has been implicated as a global regulator of virulence gene expression in a variety of bacteria, including many gastrointestinal pathogens (22, 29, 37, 40). Although many virulence and colonization factors have been identified for *C. jejuni*, relatively little is known about how the organism regulates the expression of these factors during survival in different ecological niches. Since the emergence of full genomic sequences of *C. jejuni*, it has become apparent that the organism lacks many of the global regulatory proteins that have been shown to play such a key role in gene regulation in pathogens like *Salmonella enterica* serovar Typhimurium and *E. coli*. Studies in organisms with smaller genomes, such as *Mycoplasma genitalium*, have suggested that in the absence of these global regulatory proteins, DNA supercoiling can play a dominant role in the regulation of gene expression (22). In this study, we report the key role played by DNA supercoiling in the regulation of motility and flagellar genes in *C. jejuni* and show that growth in the presence of chicken mucus can affect DNA supercoiling levels and flagellar protein expression.

Our first observation was that a direct correlation exists between resting DNA supercoiling levels and motility levels in laboratory strains with a more relaxed DNA topology associated with lower motility, while increased negative supercoiling was associated with higher motility. Due to the high level of genetic variability between *C. jejuni* strains, a number of strains from chickens and humans were tested, and the same correlation was observed. Interestingly, during the process of transformation of the reporter plasmid into different strains, occasional clones with altered motility were observed. However, each time the motility of a clone was altered, it was shown that the resting DNA topology had also been changed. This emergence of spontaneous clones with altered motility has been described previously (41). Karlyshev et al. (41) described the emergence of motility variants at frequencies of approximately 1 × 10^−3^. Recent studies have highlighted the role played by phase variation in the regulation of FlgR, FlgS, and MotA (14–16), but we were unable to find evidence of phase variation in these genes in the clones we tested. We were unable to identify the cause of variations in motility in these clones, but our data strongly suggest that the changes observed in DNA supercoiling could play a role in this phenomenon. The ability to maintain clones with higher or lower motility at a relatively high frequency could be an important mechanism in adapting to sudden changes in environmental conditions, as has been described for other bacteria (42, 43). This observation thus implicates changes in DNA supercoiling resting levels as an additional method by which the previously described regions involved in phase variation can ensure a mixed phenotypic population within *C. jejuni* populations.

The relaxation of DNA supercoiling with novobiocin was shown to result in a reduction in motility without affecting the growth of *C. jejuni* and appeared to be associated with reduced transcription of *flgR* and aberrant transcription of *flaA* and *flaB*. This was shown to lead to decreased overall flagellin protein levels and FlgR levels but, interestingly, increased levels of FlgS, which forms a two-component regulatory system with FlgR. These changes in the expression of FlgR and FlgS did not appear to be associated with regions within these genes previously shown to play a role in phase variation. Furthermore, this variation in protein levels appeared to reduce motility, but electron microscopy did not find an observable effect on flagellar structure. The effects of porcine gastric mucin on DNA supercoiling and motility were seen to be the opposite of those observed with novobiocin, with increased DNA supercoiling, increased motility, and increased FlgR production that was coupled with decreased FlgS production. These results confirm the existence of a specific supercoiling-sensitive regulon that is characterized by divergent expression of the FlgRS two-component regulatory system.

The FlgRS system has been well characterized and is critical for the regulation of flagellar genes in *C. jejuni* (9, 10, 12). However, the divergent expression of FlgR and FlgS is counterintuitive for a two-component regulatory system. It might be expected that the expression of both proteins would be coregulated if they exclusively function as partners in this regulatory system. One possibility is that each protein could interact with a protein from another two-component regulatory system. A recent study has highlighted the existence of a specificity determinant on FlgR to prevent cross talk with other histidine kinases (44), suggesting that the two proteins interact exclusively with each other. However, other studies have reported that FlgR and FlgS are phase variable, suggesting that at times it is beneficial to have altered expression of one protein of the two-component system (14, 15). Our results show that changes in DNA topology result in divergent expression of these two proteins, suggesting that this may be a method to alter motility by disrupting the stoichiometry of this novel two-component regulatory system. It would be interesting to see whether the increased levels of FlgS expression upon relaxed DNA supercoiling directly inhibit the functionality of the flagella or whether this increase leads to interaction with a second response regulator and activation of a different regulon. Further studies are required to identify the transcriptome of this supercoiling-sensitive regulon and, specifically, to dissect the molecular mechanism by which this loss of motility is mediated.

During commensal infection of the chicken, *C. jejuni* is predominantly found in the mucus layer, suggesting that this is a critical environment for the organism. Our findings suggest that growth in the presence of chicken mucus results in an increase in negative supercoiling and a characteristic increase in FlgR and decrease in FlgS protein levels. Motility is known to be important for colonization (3), and one group has described how *C. jejuni* strains can adapt to growth in the avian gastrointestinal tract and that this adaption can result in clones with higher motility (45). We propose that the levels of DNA supercoiling are affected by chicken mucus and that this in turn leads to greater motility and increases the ability of the organism to survive and colonize in greater numbers. It was particularly interesting to observe that the greatest increase in DNA supercoiling and effect on protein expression was observed with mucus from the chicken colon as opposed to mucus from the chicken cecum. Naughton et al. have reported that when tested against a panel of mucins from different species, *C. jejuni* displays a tropism for chicken mucins and a much stronger tropism for mucins from the colon than for those from the cecum (34). A previous study reported that binding to chicken mucins can inhibit the invasion of epithelial cells by *C. jejuni* and that this inhibition was greater with mucins from the colon than with mucins from the cecum (32). This increased binding to mucins from the colon may explain the greater effect on DNA supercoiling levels when grown in the presence of mucus from this site and could suggest that changes in DNA supercoiling are induced by direct binding to chicken mucins *in vivo*.

In summary, this study identifies a role for DNA supercoiling in the regulation of genes in *C. jejuni*. This supercoiling-dependent regulon appears to be characterized by divergent expression of the FlgRS two-component regulatory system and a subsequent effect on motility, which is known to be an important colonization factor. This critical role for supercoiling in the regulation of motility could be an indication that DNA supercoiling also plays a key role in the global regulation of virulence genes in *C. jejuni* and that, in the absence of more classical global regulators, DNA supercoiling lies at the apex of gene regulation in this pathogen.

## MATERIALS AND METHODS

### Bacterial strains and growth conditions.

*C. jejuni* 81-176 is a well characterized sequenced strain used widely in infection studies, and *C. jejuni* NCTC 11168 is the type strain of *C. jejuni*. Strains of *C. jejuni* originally isolated from chickens were acquired from the laboratory of Seamus Fanning, and strains of *C. jejuni* originally cultured from patient feces were acquired from the Microbiology Laboratory, Our Lady’s Hospital for Sick Children, Dublin, Ireland. All strains were cultured at 37°C on Mueller-Hinton (MH) agar (supplemented with 50 µg/ml of kanamycin when appropriate) under microaerophilic conditions (5% O_2_, 5% CO_2_, 90% N_2_) for 48 h. *Campylobacter* strains were stored at −80°C in 25% (vol/vol) glycerol and 75% (vol/vol) MH broth.

### Ethics statement.

The strains of *C. jejuni* cultured from patient feces were isolated by staff at Our Lady’s Hospital for Sick Children during routine testing for *C. jejuni* infection. These strains were stored without any patient tissue and without any link to patient information.

### Growth curves.

Bacteria cultured at 37°C on MH agar (supplemented with 50 µg/ml of kanamycin when appropriate) in microaerophilic conditions for 48 h were inoculated into MH broth to a final optical density at 600 nm (OD_600_) of 0.04. The OD_600_ was recorded at 12 h, 24 h, and 48 h. Samples were taken at each time point, and serial dilutions were plated onto MH agar. The plates were incubated for 4 to 5 days at 37°C in a microaerophilic atmosphere. All colonies were counted, and the CFU/ml were recorded. All experiments were carried out in triplicate.

### Motility plates.

Bacteria were cultured on MH agar for 48 h. Each strain was inoculated into the center of a 30-ml motility agar plate (MH broth containing 0.4% nutrient agar) in the presence or absence of novobiocin or mucin using a sterile pipette tip. Each plate was incubated upright for 48 h, after which it was photographed and the halo diameter was recorded.

### Electroporation of *C. jejuni* strains with pRY107.

The pRY107 plasmid was kindly donated by the laboratory of Patricia Guerry. The plasmid was electroporated into the laboratory strains, as well as the chicken and human isolates. Bacteria were streaked from frozen stock onto MH agar (48 h of growth). After 48 h, the bacteria were restreaked onto fresh MH agar using a heavy inoculum and were grown for 16 to 18 h. Bacteria were resuspended from the MH agar by adding 1.5 ml of MH broth. Bacteria were centrifuged for 5 min at 14,000 × *g* at 4°C. The pellet was gently resuspended in ice-cold wash buffer (9% sucrose, 15% glycerol). Bacteria were collected by centrifugation for 5 min at 14,000 × *g* at 4°C. The bacteria were washed a further 3 times in the ice-cold wash buffer. The electrocompetent cells were kept on ice for the remainder of the experiment. In a microcentrifuge tube, 50 µl of electrocompetent bacteria and up to 2 µl of DNA were combined. The bacterium-DNA mixture was added to an electroporation cuvette. The sample was electroporated at 2.5 kV, 200 Ω, and 25 µF. Immediately thereafter, the cuvette was flushed with 100-µl superoptimal broth with catabolite repression (SOC) medium. The bacteria were spread onto MH plates (without antibiotics) to allow them to recover. The plates were incubated for 5 h at 37°C under microaerophilic conditions. Bacteria were harvested by adding 1.5 ml of MH broth to the MH plate and pelleted by centrifugation for 2 min at 14,000 × *g*. The supernatant was removed, and the pellet was carefully resuspended in 100 µl of MH broth. Bacteria were plated onto MH plates containing kanamycin (50 µg/ml). The plates were incubated for 4 to 5 days at 37°C in microaerophilic atmosphere. Several colonies of each strain were picked and grown overnight in 10 ml broth, and the presence of the plasmid was confirmed by plasmid isolation using the Qiagen miniprep kit, followed by agarose gel electrophoresis of the plasmid digested with the NotI enzyme, which cuts at a single site on the plasmid.

### Chloroquine gel electrophoresis.

To determine the level of supercoiling on the bacterial genome, plasmid topoisomers of pRY107 were separated on 1% agarose gels containing Tris-borate-EDTA (TBE) (2×) with 10 µg/ml of chloroquine. Bacteria were grown for 24 h in Mueller-Hinton broth containing 50 µg/ml kanamycin, and plasmid DNA was isolated (Qiagen miniprep kit). Electrophoresis was carried out at a constant voltage of 150 V for 24 h. The running buffer contained TBE (2×) and 10 µg/ml chloroquine. Chloroquine was washed from the gel by soaking in distilled water for at least 24 h before staining with ethidium bromide (10 µg/ml).

### RNA isolation and RT-PCR/qPCR.

Total RNA was isolated from cultures in exponential-growth phase. *C. jejuni* isolates were streaked from frozen stock onto MH agar and incubated for 48 h. Bacteria were inoculated into MH broth and incubated overnight. Bacterial numbers were then equalized to 0.2 (OD_600_) and were cultured for different times according to the individual experiment. Each bacterial culture was equalized to an optical density of 1 (at OD_600_) in 1 ml, and the total RNA was isolated using Trizol reagent (Invitrogen) following the manufacturer’s instructions. Total RNA was resuspended in 40 µl of diethyl pyrocarbonate (DEPC)-treated water. The integrity of the RNA was evaluated on a 2% agarose gel. For each sample, RNA was treated with DNase in a 25-µl reaction mixture (Ambion). The RNA concentration was measured using an ND-1000 spectrophotometer. cDNA templates were synthesized by random priming 2 µg of total RNA in a 20-µl reaction mixture using the high-capacity cDNA kit (ABI). For RT-PCR, a negative control for each RNA sample was included to confirm the absence of genomic DNA. Amplification of cDNA was carried out (1 cycle of 94°C for 5 min, 15 to 30 cycles of 94°C for 30 s, 55°C for 30 s, and 72°C for 45 s, and 1 cycle of 72°C for 10 min) with the primers listed in Table 1. The PCR products were run on a 2% agarose gel. Due to the high number of genes to be tested, SYBR green was chosen as the appropriate chemistry for confirmation by qPCR analysis. qPCR was carried out in triplicate with each primer set on an ABI 7900HT fast real-time system (Applied Biosystems) using SensiFast SYBR green hi-ROX (Bioline).

**TABLE 1:** Primers used for RT-PCR^*a*^

Gene	Forward	Reverse
*rpsM*	5′ GAGTTAAGTGAAGATGAAGCAGCA 3′	5′ TGTTTTCTCAAGTCCCCTTCA 3′
*flaA*	5′ CTTAAAGGCGCTATGGCTGT 3′	5′ GAACCAATGTCGGCTCTGAT 3′
*flaB*	5′ ACACCAACATCGGTGCATTA 3′	5′ CCATCCCTGAAGCATCATCT 3′
*flgR*	5′ TATAGAAGCGGTTCGTTTGG 3′	5′ GCACGCTTAATAGCCTCGAC 3′
*flgS*	5′ TAGCGATGAAAAACGCAATG 3′	5′ CGATACGCTCCACTCTAGCAA 3′
*gyrA*	5′ TGAAGGCTCAAGAACGGCTA 3′	5′ ATCAAAATCCGTGGTTGGAA 3′
*gyrB*	5′ CGATGAAGCTATGGCAGGAC 3′	5′ TGGGTGCATATCAACAGGAA 3′
*topA*	5′ GACTTAGTGCTGGGCGTGTT 3′	5′ CTAGGGGTACAAAGGCACGA 3′

a Primers were designed to amplify regions within the open reading frame of each gene listed.

### SDS-PAGE and Western blotting.

Whole-cell lysates were prepared by culturing bacteria on MH agar for 48 h under microaerophilic conditions at 37°C. Bacteria were inoculated into MH broth and equalized to 0.04 (OD_600_). Bacterial cultures were incubated for 18 h under microaerophilic conditions at 37°C. Samples were equalized to 0.6 (OD_600_) in 1 ml to ensure equal loading of protein. Bacterial cultures were centrifuged for 5 min at 13,000 × *g*. The bacterial pellet was resuspended in 100 µl of 1× Laemmli buffer, and 10 µl of sample was separated with 5% stacking and 10% SDS-PAGE gel (100 V, constant voltage). Protein gels were stained with Coomassie brilliant blue to confirm equal loading or transferred to polyvinylidene difluoride (PVDF) membrane for immunological detection when appropriate. PVDF membranes were blocked for 1 h in 3% milk at room temperature prior to the addition of the primary antisera. For immunoblotting of the PVDF membrane, antisera against different *Campylobacter* flagellar proteins were provided by the laboratory of Patricia Guerry. Antisera against FlgR and FlgS were used at a concentration of 1:10,000, and antiserum against flagellin was used at a concentration of 1:200,000. Blots were detected by using a goat anti-rabbit antibody (1:10,000) conjugated to horseradish peroxidase, followed by detection with SuperSignal west pico/dura detection kits.

### DNA isolation and sequence analysis of regions associated with phase variation.

DNA was isolated from bacteria grown in the presence and absence of novobiocin using the previously described cetyltrimethylammonium bromide (CTAB) method of DNA isolation (46). Regions identified in previous studies as playing a role in phase variation of *flgS*, *flgR*, and *motA* were amplified using Bioline MyTaq red mix (see Table S2 in the supplemental material for a list of primers used) and sequenced using the Eurofins Mix2seq kit.

### Transmission electron microscopy.

*C. jejuni* NCTC 11168 grown on MH agar was resuspended to an OD_600_ of 0.2 and grown for 4 h in MH broth. Novobiocin was then added to each sample, and the culture was allowed to grow for a further 4 h. Bacteria were equalized to an OD_600_ of 1 and collected by gentle centrifugation. The pellet was gently resuspended in a 2% (vol/vol) glutaraldehyde solution for fixation. Fixation was completed by incubating strains for 1 h on ice. All samples were stained with 1% (wt/vol) uranyl acetate and visualized with a JOEL 1200× transmission electron microscope at 80 kV.

### Preparation of porcine mucin and chicken mucus.

Porcine gastric mucin (Sigma) was prepared at 10 mg/ml. Sterility was confirmed by plating an aliquot onto MH agar and incubating at 37°C under aerobic and microaerophilic conditions for 5 days. The intestines from three chickens obtained from a commercial enterprise (Organic Foods Co., Kildare, Ireland) were carefully separated from the offal. A transfer pipette containing phosphate-buffered saline (PBS) was used to remove any fecal matter or waste from inside the colon or cecum. The tissue was slit longitudinally and pinned back on a corkboard. A clean transfer pipette with 4 M guanidinium chloride (GdnCl) containing a cocktail of protein inhibitors (5 mM EDTA, 5 mM *N*-ethyl maleimide, 0.1-mg/ml soybean trypsin inhibitor, 10 µM benzamide, 1 mM phenylmethylsulfonyl fluoride [PMSF]) was used to remove any adherent mucus by washing the epithelium of the intestines. The solution was incubated for 15 min, and mucus was collected using a transfer pipette. An additional 4 M GdnCl was then added, and the epithelium was gently scraped to remove any remaining adherent mucus. The mucus was collected in 50-ml tubes, and samples were left in 4 M GdnCl at room temperature to disperse for 4 to 5 days on a roller. The GdnCl was removed by dialysis (8-kDa molecular mass cutoff; Fisher Scientific) in deionized water over 48 h. The colonic and cecal mucus samples were freeze-dried, and the dry weight was determined by subtracting the weight of the preweighed 50-ml tube from that of the tube containing the freeze-dried mucus. The mucus was resuspended in sterile deionized water to a final concentration of 10 mg/ml and stored at −20°C.

### Statistical analysis.

Statistical analysis was carried out using Student’s *t* test (two-tailed distribution). Statistically different values between samples possessed *P* values of <0.05. All experiments were performed in triplicate, with at least three biological replicates.

## SUPPLEMENTAL MATERIAL

Figure S1  Comparison of sequences from regions previously identified as playing a role in phase variation of FlgR, FlgS, and MotA in strain NCTC 11168 grown in the presence and absence of novobiocin. Genomic analysis revealed that growth of *C. jejuni* NCTC 11168 in the presence or absence of novobiocin had no effect on the sequence of regions previously described as playing a role in phase variation (regions highlighted in grey). Download Figure S1, TIF file, 0.1 MB

Figure S2  Comparison of sequences from regions previously identified as playing a role in phase variation of FlgR, FlgS, and MotA in clones with altered motility and resting supercoiling levels. Genomic analysis of clones A and B revealed no effect on the sequence of regions previously described as playing a role in phase variation (regions highlighted in grey). Download Figure S2, TIF file, 0.1 MB

Table S1  Primers used for RT-PCR. Primers were designed to amplify regions previously described as playing a role in phase variation for *flgR*, *flgS*, and *motA* Table S1, DOCX file, 0.05 MB
