# Supplementary figures and images for: DNA Supercoiling Regulates the Motility of Campylobacter jejuni and Is Altered by Growth in the Presence of Chicken Mucus
Source: mBio. 2016 Sep 13;7(5):e01227-16. doi: 10.1128/mBio.01227-16 (PMC5021803; doi:10.1128/mBio.01227-16)

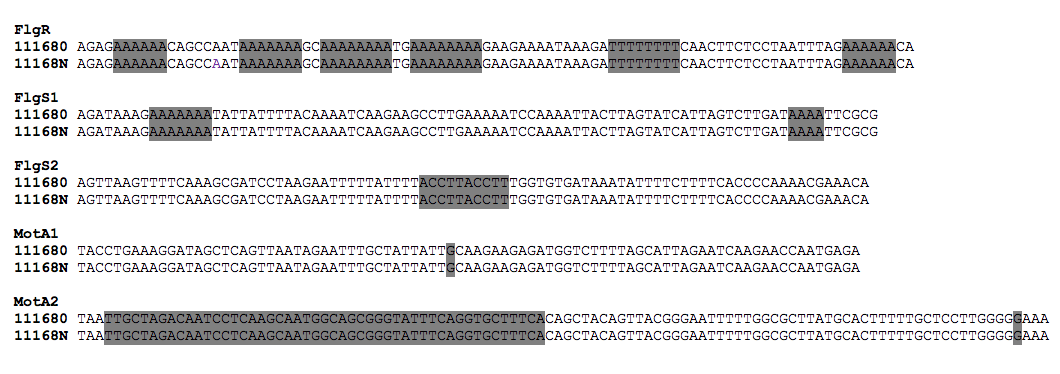

Supplement: Figure S1 — Comparison of sequences from regions previously identified as playing a role in phase variation of FlgR, FlgS, and MotA in strain NCTC 11168 grown in the presence and absence of novobiocin. Genomic analysis revealed that growth of C. jejuni NCTC 11168 in the presence or absence of novobiocin had no effect on the sequence of regions previously described as playing a role in phase variation (regions highlighted in grey). Download [file mbo004162967sf1.tif]

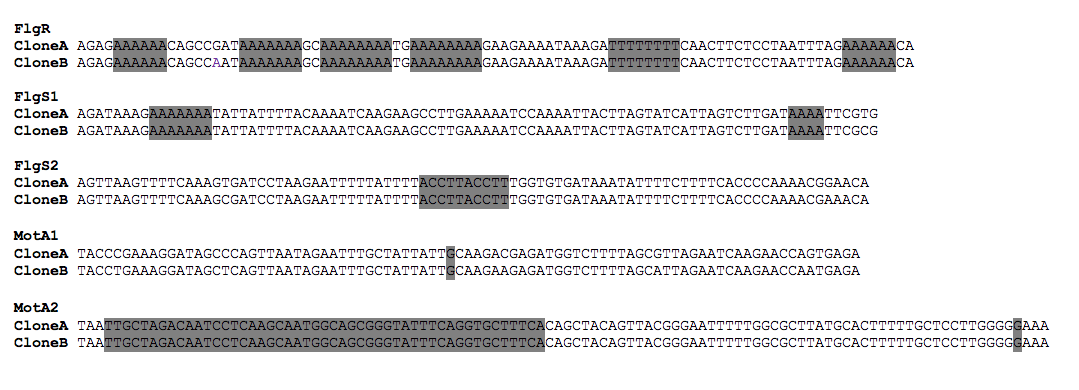

Supplement: Figure S2 — Comparison of sequences from regions previously identified as playing a role in phase variation of FlgR, FlgS, and MotA in clones with altered motility and resting supercoiling levels. Genomic analysis of clones A and B revealed no effect on the sequence of regions previously described as playing a role in phase variation (regions highlighted in grey). Download [file mbo004162967sf2.tif]
