# Supplementary material for: DNA Supercoiling Regulates the Motility of Campylobacter jejuni and Is Altered by Growth in the Presence of Chicken Mucus
Source: mBio. 2016 Sep 13;7(5):e01227-16. doi: 10.1128/mBio.01227-16 (PMC5021803; doi:10.1128/mBio.01227-16)
Supplement: Table S1 — Primers used for RT-PCR. Primers were designed to amplify regions previously described as playing a role in phase variation for flgR, flgS, and motA [file mbo004162967st1.docx]

**Table S1: Primers for analysis of phase variable regions.**

| **Gene** | **Forward** | **Reverse** |
| --- | --- | --- |
| ***flgR*** | 5’GCGGTTCGTTTGGGAGTAAA-‘3 | 5’-AATTTGCAGGAATGGCTGCC-3’ |
| ***flgS1*** | 5'-TAACTCCTTACGCGCAATGG-3' | 5'-TCTCATGTGCCAAATGAGCC-3' |
| ***flgS2*** | 5’-CAAAACTTAAAGGCAATG-3’ | 5’-tgcttcacttcttgctagag-3’ |
| ***MotA1*** | 5’-GGATCTTTCAACCATATTAGG-3’ | 5’-AGCTTTTACCATCTACAAGCA-3’ |
| ***MotA2*** | 5'-TGGAGAAACTTGCCCTACCA-3' | 5'-TGTGAAATTCTTGGATCATCGTG-3' |
